# A Reversible Color Polyphenism in American Peppered Moth (*Biston betularia cognataria*) Caterpillars

**DOI:** 10.1371/journal.pone.0003142

**Published:** 2008-09-04

**Authors:** Mohamed A. F. Noor, Robin S. Parnell, Bruce S. Grant

**Affiliations:** Biology Department, College of William & Mary in Virginia, Williamsburg, Virginia, United States of America; University of Sheffield, United Kingdom

## Abstract

Insect body color polyphenisms enhance survival by producing crypsis in diverse backgrounds. While color polyphenisms are often indirectly induced by temperature, rearing density, or diet, insects can benefit from immediate crypsis if they evolve polyphenisms directly induced by exposure to the background color, hence immediately deriving protection from predation. Here, we examine such a directly induced color polyphenism in caterpillars of the geometrid peppered moth (*Biston betularia*). This larval color polyphenism is unrelated to the genetic polymorphism for melanic phenotypes in adult moths. *B. betularia* caterpillars are generalist feeders and develop body colors that closely match the brown or green twigs of their host plant. We expand on previous studies examining the proximal cues that stimulate color development. Under controlled rearing conditions, we manipulated diets and background reflectance, using both natural and artificial twigs, and show that visual experience has a much stronger effect than does diet in promoting precise color matching. Their induced body color was not a simple response to reflectance or light intensity but instead specifically matched the wavelength of light to which they were exposed. We also show that the potential to change color is retained until the final (sixth) larval instar. Given their broad host range, this directly induced color polyphenism likely provides the caterpillars with strong protection from bird predation.

## Introduction

Phenotypic plasticity allows a single organismal genotype to display an adaptive form in diverse environments. Nijhout [1] divided phenotypic plasticity into two broad types- a basal form of absence of homeostatic mechanisms to buffer an organism's phenotype against environmental variation and a derived form that adapts an organism to particular environments. One characteristic of the latter type is that the inducing environment is often not the same as (and generally precedes) the selective environment. For instance, an organism may respond to shortening photoperiod by exhibiting a morphology or behavior to prepare for limited food availability.

Color and patterning polyphenisms in insects often fit this principle of a distinct inducing environment and selecting environment. Some color polyphenisms are induced by rearing temperature differences, as in the tomato hornworm (*Manduca quinquemaculata*) and American grasshopper (*Schistocerca americana*) [2], [3]. Patterning in desert locust *Schistocerca gregaria* nymphs is influenced by rearing density [4]. Similarly, caterpillars of the emerald moth *Nemoria arizonaria* exhibit a polyphenism in color and morphology induced by seasonal differences in diet related to materials ingested in leaves from the host plant [5], [6]. As characterized by Nijhout [1], the inducers (temperature, rearing density, or compounds within the host plant) are distinct from the presumed-adaptive induced phenotypes (match to a particular background or aposematism).

**Figure 1:**
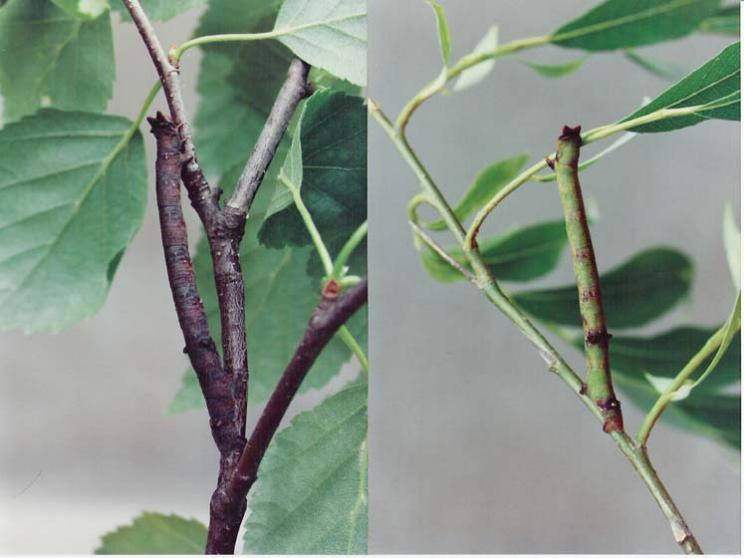
*Biston betularia* caterpillars on birch (left) and willow (right).

In other instances, developmental responses to environmental stimuli are more direct, where light intensity, reflectance, or background color induce a similar phenotype in the organism [e.g.], [7,8]. Here, we examine one such case: phenotypic plasticity in *Biston betularia* caterpillars wherein the inducer is essentially the same as the induced adaptive phenotype. Unlike the famous (or infamous among creationists) melanic vs. peppered genetic polymorphism of adult moths [9], [10], caterpillars of this species display an independent polyphenism [11] in which they resemble the color of the host tree twigs [12], [13](see Figure 1). The camouflage protects the caterpillars from predation by birds [14]. This adaptive polyphenism appears to have a large visual component, such that caterpillars raised on the same diet but with different visual cues develop to resemble the branches and twigs they encounter [11]. Hence, the visual appearance of their surroundings is the same as the induced resemblance-to-surroundings phenotype. An advantage to this type of directly induced polyphenism is that it potentially retains flexibility to changing environments: if maximally flexible, an organism will be immediately adapted to an environment that has never been encountered without any need for a selective shift in reaction norms or other developmental responses.

In this study, we re-evaluate the role of direct visual vs. indirect dietary cues in larval color polyphenisms in *Biston betularia*, examine the extent of flexibility in the plastic response, and determine whether and when the critical period allowing the formation of the adaptive polyphenism ends during development. We acknowledge that this color polyphenism may be better described as a reaction norm, but we did not examine a range of intermediate inducers to make this distinction.

## Materials and Methods

### Samples

*Biston betularia* is widespread throughout the northern hemisphere, and larvae of the North American subspecies, *B. betularia cognataria*, were used in this study. Caterpillars were produced by a breeding stock which was established from adult moths captured during the summer of 1990 at the Mountain Lake Biological Station in Virginia. This sample included no melanic adults [15], a phenotype genetically determined by an autosomal dominant allele at the “carbonaria” locus [16]. Newly hatched larvae were available in continuous supply through the experimental period.

### Setup and provisioning

Immediately following hatching from eggs, caterpillars from each brood were placed in 4-l clear plastic containers with crab-apple (*Malus sp*.) leaves for food. Within a week, they were transferred to experimental environments. All caterpillars used in different treatments of the same experiment were from the same brood or from equal numbers of different broods. Caterpillars were provisioned with fresh leaves daily throughout the experimental period, and within given treatments, diet was restricted to a single food source: crab-apple, weeping willow (*Salix babylonica*), or birch (*Betula nigra*). Leaves were cut from trees on site and brought directly to the caterpillars within an hour. In certain experiments, leaves were stripped from the branches, and in others, leaves remained attached. Except where noted, all experiments were performed in a humidified laboratory with overhead fluorescent lighting set on a 12 hour L:D cycle.

After four to five weeks of development in controlled surroundings (when in their final larval instar), caterpillars were visually evaluated for body color. Efforts to quantify color using a spectrophotometer or standardized color charts failed to produce consistent results due to the living caterpillars' movement or nonuniformity of color across the caterpillars' bodies. Given the small number of categories (see below), the most repeatable and efficient means at our disposal was scoring by the human eye. The bird's eye view of the world differs from that of humans particularly in the UV range [17], but bird prey items ranked by humans for conspicuousness on various backgrounds are also consumed in the same order when offered to birds [10], [14], suggesting that what humans see within the visible spectrum is related to what birds see. Scoring for experiments 1–5 was done by assigning individual caterpillars as “best fits” to one of four categories: 1 = bright green, 2 = green intermediate, 3 = brown intermediate, and 4 = very dark brown. Two people worked together to ensure that the scoring was consistent. Caterpillars were scored independently by the observers as they were removed from the treatment tubs (still tightly clinging to branches). Rare (∼5%) disagreements about intermediate rankings (2 and 3) were resolved by discussion, but the extreme phenotypes (1 and 4) differed qualitatively and were scored identically by both observers. While some potential observer bias cannot be completely excluded, the treatment differences in caterpillar color were effectively qualitative (Figure 1), and we are confident that any minor subjectivity does not alter our conclusions. Containers were cleaned regularly of rotten leaves, dead caterpillars, and fecal matter.

## Results

### Experiment 1: Intact host plants

To determine the extent to which *B. betularia cognataria* responds to the experimental host plants selected for this study, caterpillars were placed in two grey plastic trash barrels (128-l): one containing intact green willow branches and leaves and the other containing intact brown birch branches and leaves (Fig. 1). The tops of the barrels were covered with black chiffon cloth to keep larvae from escaping. We observed a highly significant difference between the distributions of body color phenotypes among caterpillars raised on intact willow vs. intact birch (Table 1, G = 745, P<0.001).

**Table 1:** Comparison of color distribution among caterpillars fed willow branches with intact leaves and caterpillars fed birch branches with intact leaves, or crab-apple leaves on birch vs. willow branches.

Treatment		Color Score				G-statistic	P
		A	B	C	D		
Intact leaves	Willow-fed	152	176	56	20	744.64	<0.001
	Birch-fed	0	15	69	364		
Crab-apple leaves	Willow-branches	44	120	139	124	501.7	<0.001
	Birch-branches	0	2	13	420		

Scoring was based on 1 = green, 2 = green intermediate, 3 = brown intermediate, 4 = dark brown.

### Experiment 2: Effect of branches

To begin to test the effect of color backgrounds on body color development, food was controlled. In both treatments in this experiment, caterpillars were fed crab-apple leaves from the same tree. Two yellow waste baskets (48-l) covered with white chiffon were used. In one container, green willow and bamboo branches, stripped of all leaves, were used to produce a green environment. In the other container, brown birch branches stripped of leaves were used to produce a brown environment. Again, we observed a highly significant difference in the distribution of body color phenotypes between caterpillars in the two treatments (Table 1, G = 502, P<0.001).

### Experiment 3: Artificial backgrounds

In this experiment, we simulated green and brown surroundings using colored pipe cleaners and containers, while again feeding all caterpillars on crab-apple leaves from the same tree. This approach eliminates any effect the real branches may have had on caterpillar color through tactile or chemical cues. For the two treatments, green pipe cleaners were placed in a yellow plastic wash tub (20-l), and brown pipe cleaners were placed in a brown plastic tub (20-l). Once again, we observed a significant difference between the treatments using the colored artificial branches (Table 2A, G = 214, P<0.001). Nearly 90% of the caterpillars raised in the brown environment were dark brown, while a wide range of phenotypes were observed in the green environment.

**Table 2:** Comparison of color distribution among caterpillars fed apple leaves A) on brown artificial branches in a brown tub or green artificial branches in a yellow tub, or B) in white vs. black backgrounds.

Treatment		Color Score				G-statistic	P
		A	B	C	D		
A	Green/yellow	17	51	55	15	214.3	<0.001
	Brown/brown	0	6	16	153		
B	White environment	16	6	11	4	61.26	<0.001
	Black environment	0	1	2	35		

Scoring for A) was as in Table 1, whereas B) represented shading from pale grey to black.

### Experiment 4: Reflectance or hue

In the first three experiments, the visual cues provided were attempts to simulate natural surroundings by using green or brown branches. However, caterpillars could respond either to hue, requiring color discrimination, or simply difference in background reflectance [dark vs. light], [as suggested by 7,8]. In this experiment, caterpillars were again fed crab-apple leaves stripped from branches but were now placed in a white plastic waste basket covered with white chiffon or a black plastic waste basket covered with black chiffon. The results are presented in Table 2B. In this case, however, the number 1 phenotype was not bright green but instead a pale grey, and the number 4 phenotype was nearly black rather than brown. There was also a highly significant difference in the distribution of body color phenotypes between the treatments (G = 61, P<0.001).

### Experiment 5: Effect of diet

To examine diet effects independent of color vision, experiments were conducted in total darkness: no light source was used at any time. The experiments were set up with two grey trash barrels (128-l) with black chiffon covering the tops. Lids were also placed on the barrels to further reduce ambient light from entering the experimental enclosures. One barrel received willow branches with leaves intact and the other received birch branches with intact leaves. Both groups exhibited a wide range of body color phenotypes, yet a significant difference was still noted between experiments (Table 3, G = 66, P<0.001).

**Table 3:** Comparison of color distribution among caterpillars fed willow vs. birch leaves A) raised in total darkness, or B) raised in clear containers but fed shredded leaves suspended on clear polyethylene nets.

Treatment		Color Score				G-statistic	P
		A	B	C	D		
A	Willow-fed	93	46	14	2	66.39	<0.001
	Birch-fed	11	22	38	4		
B	Willow-fed	0	9	36	16	9.79	0.008
	Birch-fed	0	6	41	47		

Scoring was as in Table 1.

To further control for possible effects of tactile cues, we fed another group of caterpillars with leaves that were stripped from branches, shredded, and suspended on clear polyethylene nets, this time in clear plastic containers in white waste baskets. A small but statistically significant difference was still observed between the treatments (Table 3, G = 10, P = 0.008).

### Experiment 6: Reciprocal transplants

**Figure 2:**
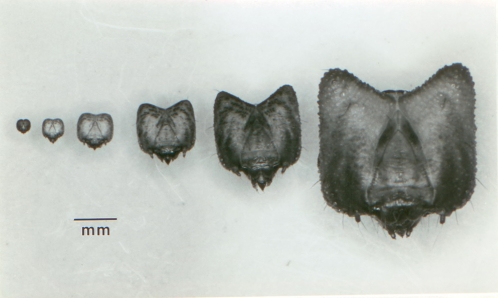
Caterpillar head sizes for instars 1–6.

We sought to determine how long during development larvae retain the ability to adjust their body colors to their surroundings. It was thus necessary to develop a means to identify the specific larval instar of caterpillars studied. Dyar [18] described six larval instars for this species and reported the mean width of caterpillar heads at the various stages, but he did not give the range of sizes within stages. We thus sought to determine whether head sizes were nonoverlapping between instars. To this end, caterpillars were reared in individual petri plates in a 20C incubator, and their sclerotized head capsules were removed after each molt and measured with an ocular micrometer to the nearest hundredth millimeter. We detected discrete, nonoverlapping gradations in head capsule size in concordance with larval instar stages (Table S1, Figure 2). Two-way analysis of variance identified significant effects of brood and sex (see Table S1), but instar determination was not confounded by this residual variance. A small number of caterpillars (4%) went through a seventh larval instar, but these caterpillars were excluded.

Caterpillars were then reared on one host, and a subset was transferred to a novel host at specific instar stages. Caterpillars in the dark brown environment were housed in 4-l clear plastic containers containing birch branches with intact leaves, wrapped in brown translucent paper, and covered with black chiffon fabric. Caterpillars in the light green environment were in similar containers but with willow branches with intact leaves, wrapped with bright green film, and topped with white chiffon fabric. Caterpillars were scored as green, intermediate, or brown. We found that fifth (penultimate larval) instar caterpillars retained the ability to change color to new surroundings after their final larval molt, whereas sixth (terminal larval) instar caterpillars could not do so (Table 4).

**Table 4:** Comparison of color distribution among caterpillars fed willow vs. birch leaves on intact branches either for their entire life or transferred at a particular larval instar.

Treatment	Color		
	Brown	Intermediate	Green
Control on birch (brown)	7	0	0
Brown to green, instar 5	0	1	11
Brown to green, instar 6	11	3	0
Control on willow (green)	0	0	6
Green to brown, instar 5	9	2	0
Green to brown, instar 6	0	1	11

## Discussion

*Biston betularia* caterpillars exhibit a color polyphenism (Figure 1) that results in striking crypsis to various types of twigs and branches. Unlike most insect polyphenisms where the environmental inducer is not the same as the selective environment, we have confirmed an earlier suggestion [11] that this polyphenism is direct, wherein background color induces a similar phenotype in the organism. Caterpillars reared on identical food substrates developed to resemble the branches they encountered. Unlike a similar polyphenism in poplar hawkmoth caterpillars [7], this polyphenism does not appear to be solely a simple response to light intensity or reflectance, but instead matches the color wavelength of the surroundings. Caterpillars also retained their ability to develop into a form cryptic to new surroundings until their final larval instar molt.

Directly induced polyphenisms are highly adaptive because they can respond to novel environments. Such polyphenisms should be most common in species which can rear on a wide range of hosts- there would be little advantage to being immediately cryptic in new environments if the species would not normally encounter new environments. *B. betularia* definitely fulfills this prediction of wide host range: caterpillars are generalists and can rear off a tremendous range of host trees and shrubs, including *Acer, Alnus, Amelanchier, Aster, Betula, Juglans, Larix, Malus, Prunus, Quercus, Rhus, Ribes,* and *Salix* [e.g., 19]. In the laboratory, first instar caterpillars climb to the top branches of their hosts, spin silk threads, and hang individually suspended underneath the leaves. This behavior in a natural setting could result in the young caterpillars being blown by the wind to different trees, and in a mixed deciduous forest, a clutch of caterpillars may be distributed over a wide variety of tree species. As such, their highly flexible directly induced polyphenism would greatly increase their cryptic potential and thus survival.

We cannot rule out the possibility that there is a dietary component to the color polyphenism: groups of caterpillars reared on shredded leaves from different hosts but in identical surroundings still exhibited a statistically significant difference in coloration. Similarly, we did observe a significant difference between caterpillars reared on different hosts in complete darkness. However, even these shredded leaves differed slightly in color and texture, so slight color or tactile cues may have contributed to the difference between the groups. We attempted to eliminate tactile cues entirely by developing agar-based synthetic foods into which birch and willow extracts were embedded. Unfortunately, too few larvae reached the final instar on these diets to permit statistical comparisons between treatments. In separate experiments, we looked for, but failed to find, an effect of texture by using smooth and rough wooden dowels to serve as twigs for caterpillars fed only on crab-apple. Clearly, direct visual stimulation has a much greater effect on larval color development than cues related to diet or surface texture.

We do not know the mechanism by which these caterpillars can change the pigment composition of their epidermal cells to create this striking crypsis. As with most other insect polyphenisms [1], expression of a novel type requires a molt in this species, and color change cannot happen within an instar [see also 11]. Hormonal changes likely stimulate the “switch” needed to change to a different phenotype, and those associated with molting, such as ecdysone or juvenile hormone, are prime candidates [20]–[22].

## Supporting Information

Table S1 Ranges and means of head sizes in millimeters for each instar and separation by sex and brood of the data for the fifth instar head capsule size
(0.04 MB DOC)
