# Supplementary material for: A Reversible Color Polyphenism in American Peppered Moth (Biston betularia cognataria) Caterpillars
Source: PLoS One. 2008 Sep 4;3(9):e3142. doi: 10.1371/journal.pone.0003142 (PMC2518955; doi:10.1371/journal.pone.0003142)
Supplement: Table S1 — Ranges and means of head sizes in millimeters for each instar and separation by sex and brood of the data for the fifth instar head capsule size (0.04 MB DOC) [file pone.0003142.s001.doc]

Table S1: Ranges and means of head sizes in millimeters for each instar and separation by sex and brood of the data for the fifth instar head capsule size.

|  |  | **N** | **Mean** | **Range** |
| --- | --- | --- | --- | --- |
| Instar 1 |  | 65 | 0.33mm | 0.31 - 0.34mm |
| Instar 2 |  | 78 | 0.55mm | 0.46 - 0.62mm |
| Instar 3 |  | 74 | 0.90mm | 0.79 - 1.06mm |
| Instar 4 |  | 74 | 1.44mm | 1.23 - 1.62mm |
| Instar 5 |  | 72 | 2.21mm | 1.94 - 2.41mm |
| Brood 1 | M | 12 | 2.08mm | 1.94 - 2.20mm |
|  | F | 17 | 2.19mm | 2.07 - 2.34mm |
| Brood 2 | M | 4 | 2.14mm | 2.05 - 2.24mm |
|  | F | 6 | 2.23mm | 2.00 - 2.33mm |
| Brood 3 | M | 6 | 2.16mm | 2.04 - 2.39mm |
|  | F | 2 | 2.27mm | 2.26 - 2.28mm |
| Brood 4 | M | 13 | 2.27mm | 2.12 - 2.37mm |
|  | F | 10 | 2.33mm | 2.24 - 2.41mm |

Analysis of variance, N=68, for size of fifth instar heads

| **Source** | **Sum of squares** | **df** | **Mean square** | **F-ratio** | **P** |
| --- | --- | --- | --- | --- | --- |
| Brood | 0.325 | 3 | 0.108 | 15.606 | <0.001 |
| Sex | 0.096 | 1 | 0.096 | 13.901 | <0.001 |
